# Rice Kefiran Ameliorates Obesity and Hepatic Steatosis Through the Change in Gut Microbiota

**DOI:** 10.3390/microorganisms12122495

**Published:** 2024-12-04

**Authors:** Takuto Kurakawa, Koudai Kani, Seita Chudan, Miyu Nishikawa, Yoshiaki Tabuchi, Kazuichi Sakamoto, Yoshinori Nagai, Shinichi Ikushiro, Yukihiro Furusawa

**Affiliations:** 1 Department of Biotechnology, Faculty of Engineering, Toyama Prefectural University, 5180 Kurokawa, Imizu 939-0398, Toyama, Japan; 2 Department of Pharmaceutical Engineering, Faculty of Engineering, Toyama Prefectural University, 5180 Kurokawa, Imizu 939-0398, Toyama, Japan; 3 Division of Molecular Genetics Research, Life Science Research Center, University of Toyama, Sugitani, Toyama 930-0194, Toyama, Japan; 4 College of Agriculture, Ibaraki University, 3-21-1. Chuo, Ami-cho, Ami-machi 300-0393, Ibaraki, Japan

**Keywords:** rice kefiran, obesity, fatty liver disease, gut microbiota, *Bacteroides*, *Alistipes*

## Abstract

Obesity is a global epidemic and a significant risk factor for various diseases. Obesity and dysbiosis are associated, drawing attention to the mechanisms that regulate the gut microbiota. In this study, we focused on the postbiotic effects of rice kefiran (Kef), a functional product of *Lactobacillus kefiranofaciens* cultured in a rice-based medium, on obesity and its complications. Although Kef has the potential to improve obesity, the underlying mechanisms remain unknown. Therefore, we aimed to elucidate the mechanisms underlying changes in gut microbiota. The administration of Kef significantly suppressed diet-induced body weight gain, reduced liver fat accumulation, and modestly improved insulin resistance. Among the gut bacteria, *Lachnospiraceae* and *Lachnoclostridium*, which were positively correlated with obesity, decreased in mice administered Kef. In contrast, *Bacteroides* and *Alistipes*, both reported to ameliorate obesity, were increased. Consistent with the changes in the gut microbiota, Kef increased fecal acetate levels, which ameliorated obesity and hepatic steatosis. Predictive metagenomic analysis suggested that Kef administration increased the abundance of KEGG orthologs, associated with carbohydrate metabolism and improvements in insulin resistance. In conclusion, Kef improves diet-induced obesity, hepatic steatosis, and insulin resistance by regulating the gut microbiota’s composition.

## 1. Introduction

Obesity is a severe public health issues, with 57.8% of the world’s population estimated to be overweight or obese by 2030 [1]. This condition is closely linked to metabolic disorders such as type 2 diabetes and hepatic steatosis [2,3]. Growing evidence suggests that an imbalance in gut microbiota, also known as dysbiosis, contributes to obesity. Notably, an increase in the proportion of Firmicutes (recently reclassified into Bacillota) and a decrease in Bacteroidetes (recently reclassified into Bacteroidota) have been shown to enhance the efficacy of energy absorption from food, thereby promoting obesity [4,5]. Therefore, there is growing interest in food components that can lower the Firmicutes/Bacteroidetes ratio (F/B ratio) as a preventive measure against obesity.

Functional food components, including probiotics and postbiotics, have attracted significant attention for their ability to modulate gut microbiota and prevent obesity [6,7,8,9,10]. Although probiotics confer various health benefits, challenges such as strain specificity, stability, and the risk of bacterial translocation into tissues or the bloodstream limit their broader application [11]. In contrast, postbiotics, defined as the “preparation of inanimate microorganisms and/or their components that confers health benefits to the host” [12,13], provide a safer and more stable alternative to probiotics. Some studies have reported that the administration of inactivated bacteria has beneficial effects comparable to or surpassing those of viable bacteria [14,15,16,17].

Here, we focused on the postbiotic effects of rice kefiran (Kef), a product of the cultivation of *Lactobacillus kefiranofaciens,* a lactic acid bacterium found in kefir. Kefir is a traditional fermented product made from cow or goat milk and is consumed in the long-lived region of the Caucasus. This fermentation process involves the lactobacilli and yeast present in kefir grains, resulting in the production of alcohol. In contrast, Kef is a concentrate derived from a monoculture of *Lactobacillus kefiranofaciens* in a rice-based medium. Unlike traditional kefir, Kef is alcohol-free, making it suitable for children and individuals with alcohol sensitivity. Kef has been shown to reduce blood cholesterol levels in both rat and rabbit models [18,19,20]. In addition, Kef lowered blood glucose levels in mice developing spontaneous type 2 diabetes. These results suggest a potential application of Kef as a postbiotic for preventing metabolic disorders; however, its anti-obesity effects and underlying mechanisms are yet to be fully elucidated.

In this study, we aimed to investigate the potential of Kef to mitigate diet-induced obesity and hepatic steatosis by modulating gut microbiota and its metabolites, providing new insights into its applications as a postbiotic.

## 2. Materials and Methods

### 2.1. Animals and Treatments

C57BL/6J mice (male, 4 weeks old) were purchased from Japan SLC (Hamamatsu, Japan). The mice were maintained under specific pathogen-free conditions and a 12 h light–dark cycle in the animal facility of Toyama Prefectural University, with free access to water and food. Temperature (22–26 °C) and humidity (50–55%) were controlled, and cages were cleaned weekly to ensure hygiene. After a one-week acclimation period, the mice were fed a high-fat diet (HFD; 60% fat; Research Diet, New Brunswick, NJ, USA) for 17 weeks. Rice kefiran (Daiwa Pharmaceutical Co., Ltd., Tokyo, Japan), containing inactivated *L. kefiranofaciens* (8 × 10^8^ cells/g) and kefiran (5 mg/g), an exopolysaccharide biosynthesized by these bacteria, was suspended in saline solution and orally administered during the period of high-fat feeding. In this study, mice were allocated to four groups: orally administered saline (CT) or three concentrations of rice kefiran diluted in saline (10, 50, and 100 mg/kg). Randomization was performed to ensure an equal distribution of body weight across groups at the start of the study. Body weight and food intake were measured weekly to monitor health and well-being. Saline or rice kefiran suspensions were prepared in a volume of 100 μL and administered 5 times per week throughout the HFD period. Animal care protocols were approved by the Animal Experiment Ethics Committee of Toyama Prefectural University (approval no. R3-13, approved on 13 May 2021).

### 2.2. Insulin Tolerance Test

The insulin tolerance test was performed as previously described [21]. The mice were kept under fasting for 3 h prior to the test, which was conducted 16 weeks after HFD feeding and Kef administration. The test was conducted following the intraperitoneal administration of 1 unit/kg insulin (Humulin R, 100 U/mL; Eli Lilly, Kobe, Japan). Blood was collected from the caudal vein at specific time points after administration. Blood glucose levels were measured using a StatStrip Xpress glucometer (Nova Biomedical, Boston, MA, USA).

### 2.3. Histological Analysis

Mice were euthanized 17 weeks after the start of HFD feeding and Kef administration to collect liver samples for further analysis. Liver sections were fixed in Mildform R 10 N (3.7–4.3% formaldehyde, Fujifilm Wako, Osaka, Japan) and prepared in the Cell Technology Laboratory (Sapporo, Japan) from paraffin-embedded livers. The deparaffinized sections were stained with hematoxylin–eosin (H&E). The lipid droplet areas were measured using ImageJ software Ver.1.54 (National Institutes of Health, Bethesda, MD, USA).

### 2.4. Isolation of Fecal DNA

Fecal samples were collected 8 weeks after Kef administration. Feces were collected from mice, immediately frozen in liquid nitrogen, and stored at −80 °C to minimize the change in microbiota composition [22]. DNA was extracted from a small amount of feces (≤50 mg) using the ZymoBIOMICS DNA Miniprep kit (Zymo Research, Irvine, CA, USA) following the manufacturer’s protocol. Bead-beating was performed for 20 min using a Disruptor Genie instrument (Scientific Industries, Bohemia, NY, USA) at maximum speed. The DNA concentration was quantified using the QuantiFluor ONE dsDNA System and Quantus Fluorometer (Promega, Madison, WI, USA).

### 2.5. 16S rRNA Sequencing and Metagenomic Functional Prediction

*16S* rRNA sequencing was performed using the MiSeq Reagent Kit according to the manufacturer’s instructions (Illumina, San Diego, CA, USA). The *16S* rRNA reads were analyzed using Quantitative Insight into Microbial Ecology 2 (QIIME2) Ver.2023.5 (Caporaso Lab, Northern Arizona University, Flagstaff, AZ, USA). Primers targeting the V3–V4 variable regions of the *16S* rRNA gene were selected. The Cutadapt plugin was used to trim the primer region (forward, 17 bases; reverse, 21 bases) from the raw sequences, followed by joining the paired-end reads (forward, 285 bp; reverse, 215 bp). Amplicon sequence variants were constructed from the processed reads using the DADA2 algorithm. To perform α- and β-diversity analyses, a diversity core-metrics phylogenetic analysis was used for 10,000 reads set at the sampling depth. Furthermore, feature classifiers, classify-sklearn, and SILVA138 databases were used to assign the taxonomy. PICRUSt2 Ver.2023.2 was utilized to predict the metagenomic functional composition [23].

### 2.6. Gas Chromatography (GC) Analysis of Fecal SCFAs

Fecal samples for the GC analysis were collected 8 and 12 weeks after Kef administration. GC was used to measure fecal SCFAs, as previously described [24]. In summary, wet feces were combined with 10 volumes of water and mixed with a toothpick to extract butyrate, valerate, acetate, and propionate from the cecum. As an internal standard, heptanoic acid was added to the supernatant after transferring to fresh tubes. Boiling stones, NaOH, pyridine, and isobutanol were added to the supernatant for derivatization using Agilent Technologies (Santa Clara, CA, USA) methods. Hexane was used to extract the derivative, and an Agilent 7820A instrument with a flame ionization detector and a DB-WAX Ultra Inert column (30 m × 250 μm × 0.5 μm) was used for GC. Helium was used as the carrier gas at a flow rate of 1 mL/min. The initial oven temperature of 40 °C was maintained for 5 min and then increased to 250 °C at a rate of 10 °C/min. The injected volume was 1 μL, and the split ratio was 5:1.

### 2.7. RNA Isolation and cDNA Preparation

Total RNA was extracted from the liver tissue and epididymal white adipose tissue (eWAT) using a NucleoSpin RNA Mini kit (TaKaRa, Shiga, Japan) following the manufacturer’s instructions. Total RNA (≤1 μg) was reverse-transcribed using the ReverTra Ace qPCR RT Master Mix (TOYOBO, Osaka, Japan).

### 2.8. Reverse Transcription–Quantitative PCR (RT-qPCR)

RT-qPCR was performed using cDNA (4 μL/reaction), THUNDERBIRD Next SYBR qPCR Mix (10 μL/reaction; TOYOBO), and a CFX Connect Real-Time System (Bio-Rad Laboratories, Hercules, CA, USA), according to the manufacturer’s instructions. The Ct values of the genes were normalized to those of *Rpl13* to calculate the relative expression levels using the ∆Ct method. The primer sequences used for qPCR are listed in Table S1.

### 2.9. Western Blotting

Protein extraction from the colonic tissue, SDS-PAGE, and transfer to PVDF membranes were performed as previously described [21]. PVDF membranes with proteins were incubated with primary antibodies, claudin-1 monoclonal antibody (mAb; Santa Cruz Biotechnology, Dallas, TX, USA) or β-Actin mAb (Fujifilm), and secondary antibody HRP-conjugated anti-mouse or anti-rabbit IgGs (Cell Signaling, Danvers, MA, USA). Protein expression levels were visualized using an enhanced chemiluminescence detection system (Merck, Darmstadt, Germany), and images were captured using an LAS-500 luminescent image analyzer (FujiFilm).

### 2.10. Statistical Analysis

Values are expressed as the mean ± standard error (SE). Student’s *t*-test, Welch’s *t*-test, or the Mann–Whitney U test were used to compare two groups. Differences were considered statistically significant at *p* < 0.05. For multiple comparisons of the *16S* rRNA amplicon sequencing data, statistical values (q-values) were calculated using Welch’s *t*-test, followed by Benjamini and Hochberg’s false discovery rate correction. The threshold for statistical significance was set at *p* < 0.05.

## 3. Results

### 3.1. Effect of Kef on HFD-Induced Obesity and Insulin Resistance

**Figure 1:**
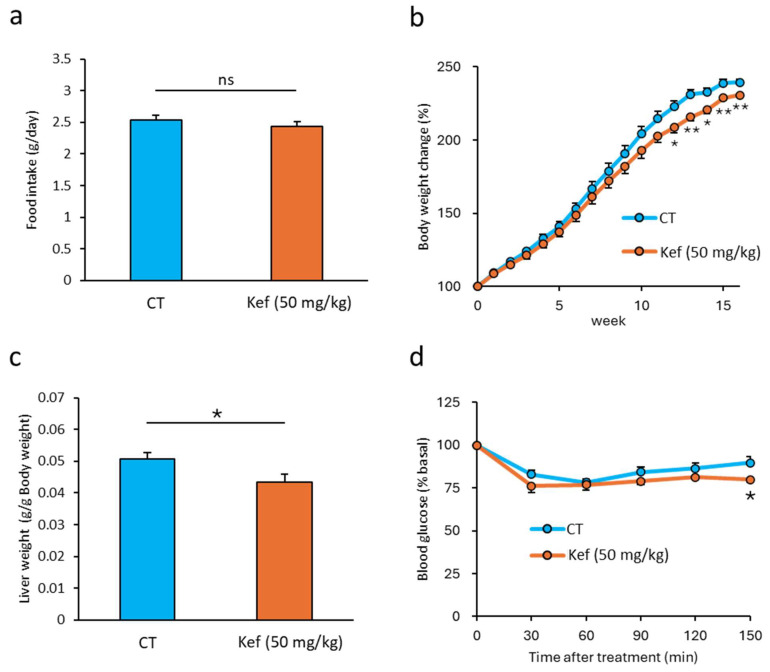
Effect of Kef on HFD-induced obesity. (**a**) Food intake (g/day) measured weekly during the experimental period. (**b**) Body weight change (percentage relative to initial weight) over 16 weeks of high-fat diet feeding (*n* = 9). (**c**) Liver weight (g) measured at 17 weeks of high-fat diet feeding (*n* = 9 or 10) and (**d**) insulin resistance evaluated by insulin resistance test; blood glucose levels were measured at specific time points (0, 30, 60, 90, 120, and 150 min) after insulin injection (*n* = 9). Data are presented as the values relative to that at 0 min (pre-injection). Values and error bars are mean ± SE. * *p* < 0.05; ** *p* < 0.01 versus HFD; ns, not significant. HFD, high-fat diet; Kef, rice kefiran.

To examine the effect of Kef on diet-induced obesity under our breeding conditions, we administered Kef to mice fed on an HFD. Although there was no difference in food consumption (Figure 1a and Figure S1c), significant reductions in body weight were observed from week 12 in the Kef (50 mg/kg) group and week 15 in the Kef (100 mg/kg) group compared with that in the CT group (Figure 1b and Figure S1b). In addition, mice that were administered Kef at 50 mg/kg were associated with reduced liver weight gain (Figure 1c). Kef administration also improved insulin resistance; however, statistical significance was observed only 150 min after insulin injection (Figure 1d).

### 3.2. Effect of Kef on HFD-Induced Hepatic Steatosis and Gene Expressions in the Liver

**Figure 2:**
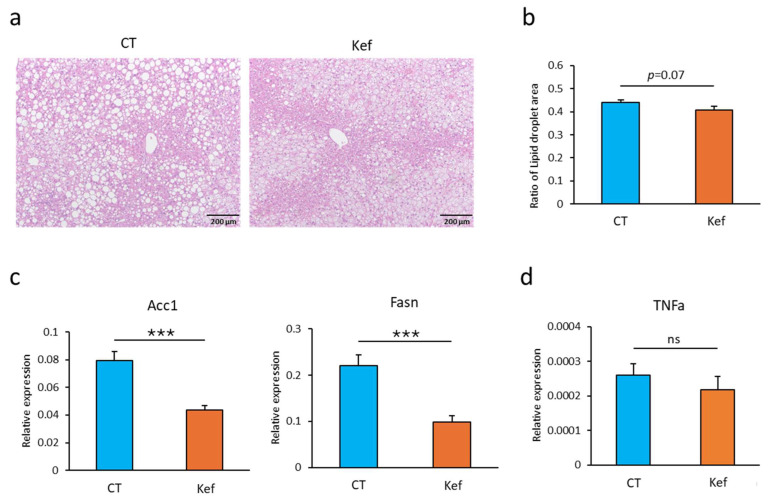
Effect of Kef on HFD-induced hepatic steatosis and gene expressions in the liver. (**a**) Representative images of liver sections stained with H&E showing lipid droplet accumulation in hepatocytes. Scale bars = 200 μm. (**b**) Quantification of lipid droplet areas (% of the total liver area) in 17–19 randomly selected locations in each group using ImageJ software (2 locations were photographed per individual). (**c**) RT-PCR analysis of genes involved in lipid synthesis (*n* = 8 or 9). (**d**) RT-PCR analysis of Tnf gene (encoding TNFα) (*n* = 8–10). Values and error bars indicate mean ± SE. *** *p* < 0.001; ns, not significant.. HFD, high-fat diet; Kef, rice kefiran.

Given that Kef (50 mg, unless otherwise specified) ameliorated HFD-induced liver weight gain, we analyzed liver fat accumulation between the two groups. Image analysis of liver sections revealed that Kef administration mitigated HFD-induced hepatic steatosis (Figure 2a,b). Furthermore, we performed RT-PCR analysis of the genes related to fatty acid synthesis in the liver. Consistent with the decrease in liver fat accumulation, the expression levels of *Acc1* and *Fasn*, which are genes related to lipid synthesis, were lower in the Kef-treated group than in the control group (Figure 2c). However, expression levels of *Ppara* (encoding PPARα), a gene associated with lipid oxidation, and those of *Ffar2* and *Ffar3* encoding the short-chain fatty acid receptor, were comparable between the two groups (Figure S2a,b) [25]. Furthermore, we analyzed the expression of *Tnf* (encoding TNFα), an inflammatory mediator that leads to insulin resistance [26]. Kef administration slightly decreased the expression level of Tnf in the liver (Figure 2d); however, the difference between the two groups was not statistically significant.

### 3.3. Effect of Kef on Gut Microbiota

**Figure 3:**
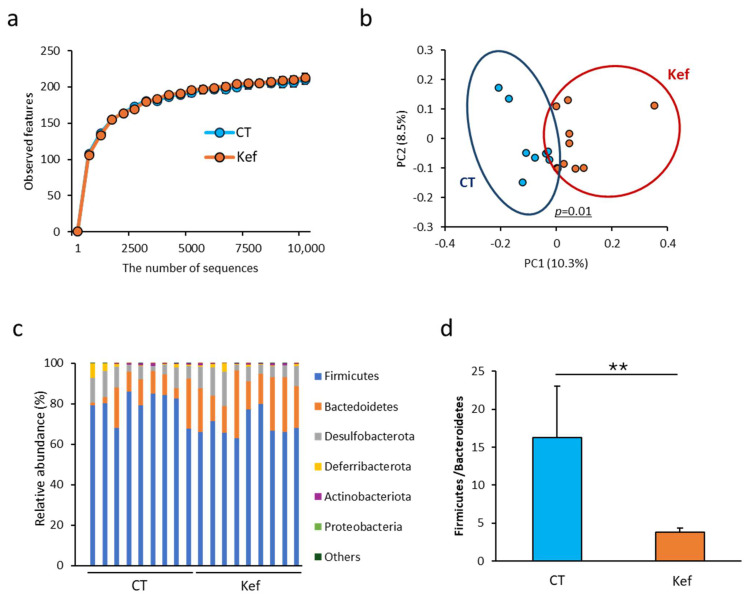
Effect of diet and rice kefiran on gut bacteria composition. (**a**) α-diversity (observed features) representing the richness of gut bacterial species within samples (*n* = 9). (**b**) β-diversity (Jaccard distance) representing the similarity between gut bacterial communities across samples (*n* = 9). The X-axis (PC1) and Y-axis (PC2) represent the first and second principal components, respectively. The percentages indicate the proportion of total variance in the dataset explained by each principal component. Each dot represents a single sample, and the clustering reflects similarities or differences in gut microbiota composition among groups. (**c**) Relative abundance (%) of bacterial phyla in the gut microbiota (*n* = 9). (**d**) Ratio of Firmicutes to Bacteroidetes (F/B ratio) (*n* = 9). Values and error bars indicate mean ± SE. ** *p* < 0.01; ns, not significant. HFD, high-fat diet; Kef, rice kefiran.

Next, we performed *16S* rRNA sequencing of fecal DNA to analyze the changes in the gut microbiota resulting from Kef administration. Although the α-diversity values, which reflect the richness and evenness of bacterial species within the population, were comparable between the control and Kef-administered mice, there was a significant difference in β-diversity values, which indicates the similarity score between populations (Figure 3a,b and Table S2) [27]. In the taxonomic analysis of the microbiota at the phylum level, the F/B ratio significantly decreased in the Kef-administered group (Figure 3c,d).

**Figure 4:**
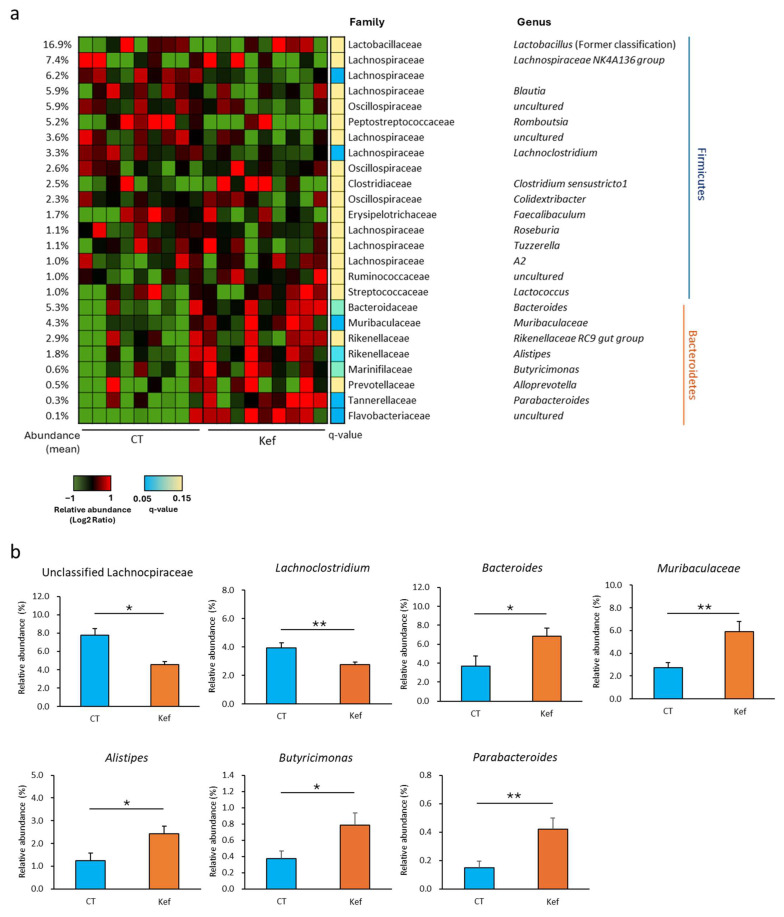
Effect of Kef on the gut microbiota’s composition at the genus level. (**a**) Change in the relative abundance of shared bacteria taxa in the gut microbiota of HFD-fed mice administered Kef (*n* = 9). Taxa with a mean abundance of more than 1% Firmicutes and 0.1% Bacteroidetes were included in the analysis. (**b**) The relative abundance of representative bacterial genera significantly affected by Kef administration. Values and error bars indicate mean ± SE. * *p* < 0.05; ** *p* < 0.01.

Further analysis of the gut microbiota’s composition at the genus level revealed that unclassified *Lachnospiraceae* and *Lachnolostridium*, classified as Firmicutes, were underrepresented in mice administered Kef. In contrast, *Bacteroides*, Muribaculaceae, *Alistipes*, *Butyricimonus*, and *Parabacteroides*, classified as Bacteroidetes, were significantly over-represented in mice administered Kef (Figure 4a,b).

### 3.4. Effect of Kef on Fecal SCFAs,

**Figure 5:**
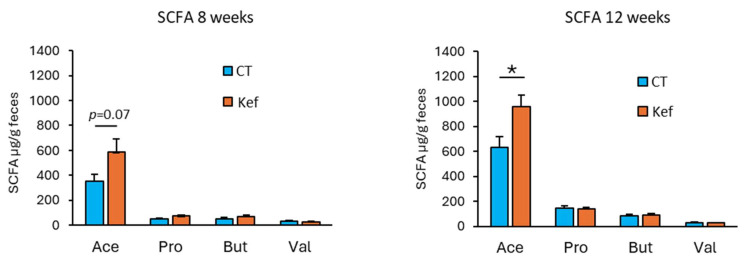
Effect of Kef on fecal SCFAs levels. Fecal samples were collected at 8 or 12 weeks after Kef administration (*n* = 9). The concentrations of four SCFAs were measured using gas chromatography. Values and error bars indicate mean ± SE. * *p* < 0.05. Ace, acetate; Pro, propionate; But, butyrate; Val, valerate. CT, saline group; Kef, rice kefiran.

*Bacteroides* and *Muribaculaceae* ameliorate obesity and fatty liver disease by producing SCFAs, particularly acetate [28,29,30]. Following the changes in gut microbiota composition, we conducted GC analysis of fecal SCFAs in Kef-administered mice. As expected, fecal acetate levels tended to increase at 8 weeks and significantly increased at 12 weeks in mice administered Kef (Figure 5). In contrast, fecal propionate slightly increased in the Kef group at 8 weeks but was comparable at 12 weeks between the two groups. Similarly, fecal butyrate also slightly increased at 8 and 12 weeks after Kef administration; however, this increase was not statistically significant.

### 3.5. Effect of Kef on Intestinal Barrier Function and Adipose Tissue Inflammation

**Figure 6:**
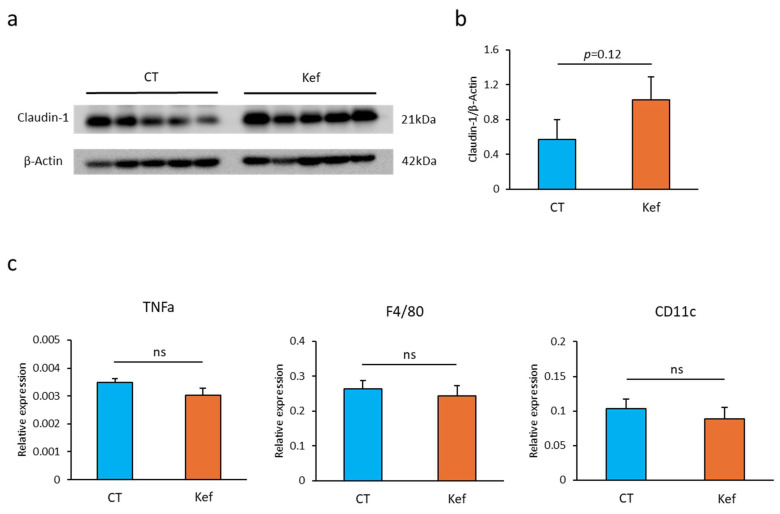
Effect of Kef on claudin-1 expression and gene expressions in adipose tissue. (**a**) Representative Western blot image showing the effect of Kef on claudin-1 protein expression in colonic tissue (*n* = 5). (**b**) Quantification of claudin-1 protein expression normalized with β-actin. Band intensities were measured using ImageJ software. (**c**) RT-PCR analysis of genes associated with inflammation (TNFa), pan-macrophage marker (F4/80), and M1 macrophage marker (CD11c) in epididymal white adipose tissue (eWAT; *n* = 8–10 per group). Values and error bars indicate mean ± SE. ns, not significant. CT, saline group; Kef, rice kefiran.

Given that changes in gut microbiota composition resulting from an HFD disturb intestinal barrier function, leading to adipose tissue inflammation and insulin resistance, we evaluated the expression of claudin-1, a crucial tight junction protein for intestinal barrier function [31,32], in mice administered Kef. Western blot analysis revealed that Kef administration increased claudin-1 expression in the colonic tissue; however, this increase was not statistically significant (Figure 6a,b).

Consistent with the claudin-1 expression levels, Kef administration slightly, but not significantly, suppressed the expression of *Tnf*, *Adrge1* (encoding F4/80, a pan macrophage marker), and *Itgax* (encoding CD11c, an inflammatory M1 macrophage in adipose tissue) (Figure 6c).

### 3.6. Effect of Kef on the Abundance of Functionally Predicted Metagenomes

**Figure 7:**
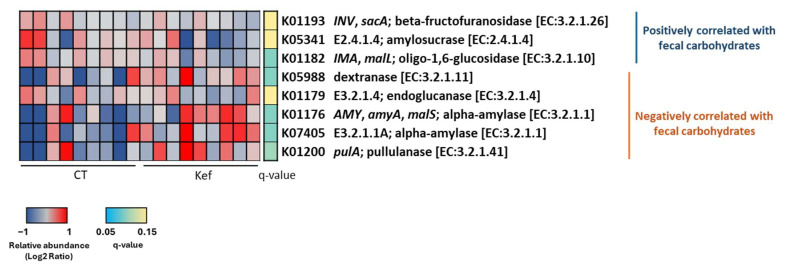
Effect of Kef on the predicted functional metagenomes of gut bacteria. Relative abundances of predicted functional metagenomes associated with fecal carbohydrates metabolism pathway in gut bacteria, analyzed using PICRUSt2 (*n* = 9 per group). Pathway predictions were based on 16S rRNA gene sequencing data. CT, saline group; Kef, rice kefiran.

Intestinal barrier function and adipose tissue inflammation alone may not adequately explain the mechanism by which Kef alleviates insulin resistance. Therefore, we conducted a predictive functional metagenomic analysis of fecal DNA using PICRUSt2 to identify gut bacteria-derived genes potentially involved in insulin resistance because a previous study reported that *Bacteroides* and *Alistipes* improve insulin resistance by reducing fecal carbohydrate concentrations through their extracellular enzymes [33]. In this study, we found that the K01182 oligo-1,6-glucosidase, which is positively correlated with the fecal carbohydrate concentration, was significantly decreased in the fecal DNA of Kef-treated mice (Figure 7). In contrast, K05988 dextranase, K01176 alpha-amylase, and K07405 alpha-amylase, which were negatively correlated with the fecal carbohydrate concentration, were significantly increased following Kef administration (Figure 7).

## 4. Discussion

The present study demonstrates that Kef administration significantly ameliorated metabolic disorders, including obesity, hepatic steatosis, and insulin resistance. These beneficial effects are closely associated with changes in gut microbiota composition, elevated levels of SCFAs (particularly acetate), and the downregulation of lipogenic genes in the liver.

The modulation of gut microbiota may underlie the beneficial effects of Kef on metabolic disorders, as non-viable microbial cells or cell constituents have been shown to improve metabolic health by altering the gut microbiota’s composition. For example, pasteurized *Akkermansia muciniphila* and its extracellular vesicle have been reported to prevent diet-induced obesity by an underlying mechanism involving an increase in *Veillonella* spp. and *Alistipes* spp. [34]. In addition, inactivated *Bifidobacterium animalis* increased the relative abundance of Bacteroidaceae and Rikenellaceae while reducing Clostridiaceae in Wistar rats, which correlated with decreased serum cholesterol and HDL levels [35]. Given that the primary component of Kef is inactivated *Lactobacillus kefiranofaciens*, its antimetabolic properties are likely attributable to alterations in the gut microbiota. In this study, Kef administration decreased the relative abundance of Firmicutes phylum, including unclassified Lachnospiraceae and *Lachnoclostridium* (Figure 3d and Figure 4b), which are positively correlated with obesity [36,37,38], while simultaneously increasing the relative abundance of Bacteroidetes phylum, including *Bacteroides*, *Alistipes*, and *Parabacteroides* (Figure 4b), which are associated with anti-obesity effects [39,40,41]. These results suggest that the change in the gut microbiota is responsible, at least in part, for the anti-obesity effect of Kef.

Among the predominant bacteria in Kef-administered mice, *Bacteroides* and *Muribaculaceae* ameliorate fatty liver disease as well as obesity by producing SCFAs, particularly acetate [28,29,30]. Acetate alleviates liver fat accumulation by binding to SCFA receptors expressed in the liver, such as G protein-coupled receptors GPR41/43 [42]. A previous study demonstrated that SCFA supplementation failed to decrease *Acc1* and *Fasn* expression in mice deficient in GPR41 or GPR43, indicating that GPR41/43 signaling plays a critical role in downregulating these lipogenic genes [43]. Therefore, the activation of GPR41/43 by elevated acetate, which involves an increase in acetate-producing bacteria, may be responsible for reduced body weight and liver fat accumulation in Kef-administered mice. However, this hypothesis must be confirmed using genetically engineered mice deficient in GPR41/43.

Meanwhile, elevated acetate levels alone may not account for the alleviation of insulin resistance by Kef administration, although previous studies have demonstrated that luminal SCFAs upregulate claudin-1 expression, leading to the restoration of intestinal barrier function and amelioration of insulin resistance in a mouse model of diabetes [44,45,46]. In this study, Kef administration slightly upregulated the expression of claudin-1; however, the downregulation of inflammatory gene expression in adipose tissue, which may result from the recovered intestinal barrier function, was limited in the Kef-administered group (Figure 6). These results suggest that the effect of Kef on intestinal barrier function and adipose tissue inflammation likely played a minor role in improving insulin resistance.

On behalf of intestinal permeability, another possible mechanism underlying the improvement in insulin resistance may involve the consumption of carbohydrates by specific bacteria in the Kef-administered mice. A previous study combining metabolome and shotgun metagenome analyses for approximately 300 healthy individuals demonstrated a correlation between increased serum monosaccharide levels, fecal monosaccharide levels, and the abundance of KEGG orthologs predicted to function as extracellular enzymes [33]. Among these orthologs, K01193 (beta-fructofuranosidase), K05341 (amylosucrase), and K01182 (oligo-1,6-glucosidase), found in *Dorea* and *Blautia,* are extracellular glucosidases that decompose dextrin and sucrose into glucose and fructose. These orthologs were positively associated with both fecal carbohydrate levels and insulin resistance. In contrast, K01176 (alpha-amylase) and K07405 (alpha-amylase), found in *Bacteroides* and *Alistipes,* are enzymes related to starch decomposition and were negatively correlated with fecal carbohydrate levels and insulin resistance. In addition, K01179 (endoglucanase), K01200 (pullulanase), and K05988 (dextranase), also identified in *Bacteroides* and *Alistipes,* were negatively correlated with fecal carbohydrate levels and insulin resistance. In this study, the metagenomic prediction analysis of fecal DNA revealed that Kef altered the abundance of KEGG orthologs toward a reduction in fecal carbohydrates (Figure 7), likely due to a decrease in *Blautia* and an increase in *Bacteroides* and *Alistipes* (Figure 4). Although we cannot demonstrate a direct relationship between fecal carbohydrates and insulin resistance due to technical limitations, Kef may improve insulin resistance, possibly through changes in carbohydrate metabolism by gut bacteria.

In conclusion, we found that the administration of Kef ameliorated metabolic disorders in HFD-fed mice by altering the gut microbiota’s composition and their metabolites. These findings highlight the potential of Kef as a postbiotic for mitigating obesity and related metabolic disorders. However, this study has several limitations that should be acknowledged. First, the absence of baseline data on the fecal microbiota prior to the HFD and kefiran treatment limits a comprehensive understanding of changes in gut microbiota throughout this study. Second, the small sample size in each experimental group reduces the statistical power and may limit the generalizability of the findings. Furthermore, while murine models provide valuable insights into the mechanisms of gut microbiota interactions and metabolic regulation, one must be cautious when extrapolating these results to humans due to significant differences in gut microbiota composition and physiological responses between mice and humans. Future studies should address these limitations by collecting baseline microbiota data, increasing sample sizes, and validating findings in human clinical trials. In particular, human studies are essential to elucidate the effects of Kef on gut microbiota and obesity and its potential application as a postbiotic.

## Data Availability

The original contributions presented in this study are included in the article/Supplementary Materials. Further inquiries can be directed to the corresponding author.

## Supplementary Materials

The following supporting information can be downloaded at: https://www.mdpi.com/article/10.3390/microorganisms12122495/s1, Figure S1: Effect of rice kefiran (Kef) at a dose of 10 and 100 mg/kg on HFD-induced obesity; Figure S2: Effect of Kef on expression of gene involved in lipid oxydation and SCFA receptors in the liver; Table S1: Primer sequences for qPCR; Table S2: α-diversity and statistical values.
