# Supplementary material for: Rice Kefiran Ameliorates Obesity and Hepatic Steatosis Through the Change in Gut Microbiota
Source: Microorganisms. 2024 Dec 4;12(12):2495. doi: 10.3390/microorganisms12122495 (PMC11728449; doi:10.3390/microorganisms12122495)
Supplement: Supplementary file 1 [file microorganisms-12-02495-s001.zip › microorganisms-3358940-supplementary.pdf]

Table S1. Primer sequences for qPCR.

Target gene or bacteria	Direction	Sequence (5' to 3')
**Rpl13a**	Forward	CCATTGTGGCCAAGCAGGTA
	Reverse	CTCGGGAGGGGTTGGTATTC
**Tnf (TNFα)**	Forward	TAGCCCACGTCGTAGCAAAC
	Reverse	GCAGCCTTGTCCCTTGAAGA
**Adrge1 (F4/80)**	Forward	CTGGGATCCTACAGCTGCTC
	Reverse	AGGAGCCTGGTACATTGGTG
**Itgax (CD11c)**	Forward	CTGGATAGCCTTTCTTCTGCTG
	Reverse	GCACACTGTGTCCGAACTCA
**Ppara (PPARα)**	Forward	AGAGCCCCATCTGTCCTCTC
	Reverse	ACTGGTAGTCTGCAAAACCAAA
**Acc1**	Forward	ATGTTGAGACGCTGGTTTGTAG
	Reverse	TCTTCCTCTGTCAGTTGCTTCT
**Fasn**	Forward	GGATGTCAACAAGCCCAAATAC
	Reverse	GAGGAGAAGGCCACAAAGTAG
**Ffar3 (GPR41)**	Forward	GTGACCATGGGGACAAGCTTC
	Reverse	CCCTGGCTGTAGGTTGCATT
**Ffar2 (GPR43)**	Forward	GGGATCTGGGTCACATGCTTAT
	Reverse	ATGTCAGACAGACGGGTACCAA

Table S2. α-diversity and statistical values.

	CT	Kef (50 mg/kg)	H	p-value	q-value
Shannon	5.77±0.117	5.734±0.104	0.096	0.757	0.757
Obeseved_features	208.78±7.14	211.11±5.27	0.160	0.689	0.689
Faith_pd	15.47±0.598	15.61±0.319	0.563	0.453	0.453
Evenness	0.749±0.011	0.743±0.012	0.096	0.757	0.757
Chao1	222.9±8.07	226.5±6.83	0.125	0.723	0.723

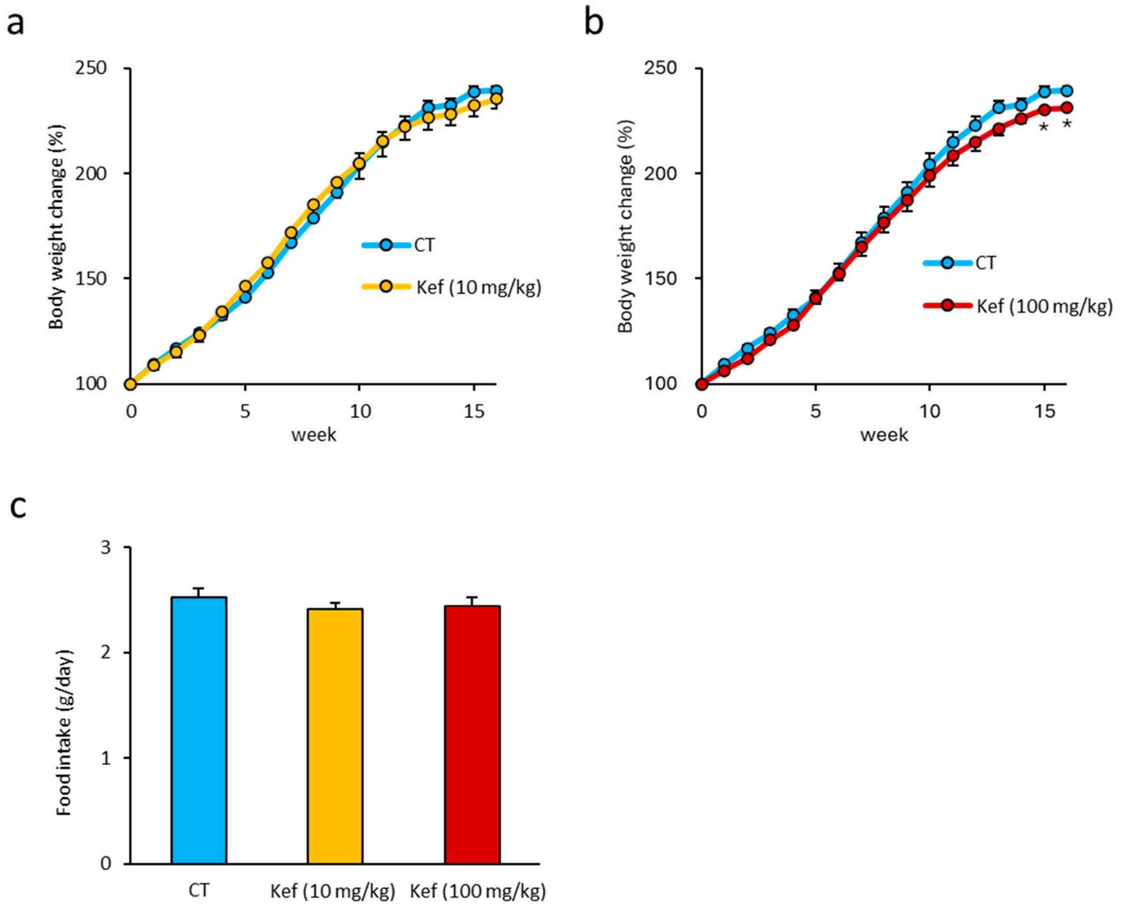

Figure S1. Effect of rice kefiran (Kef) at a dose of 10 and 100 mg/kg on HFD-induced obesity.**(a)** Effect of Kef at a dose of 10 mg/kg and **(b)** 100 mg/kg on body weight change (n = 9). **(c)** Effect of Kef on food intake. HFD, high-fat diet.

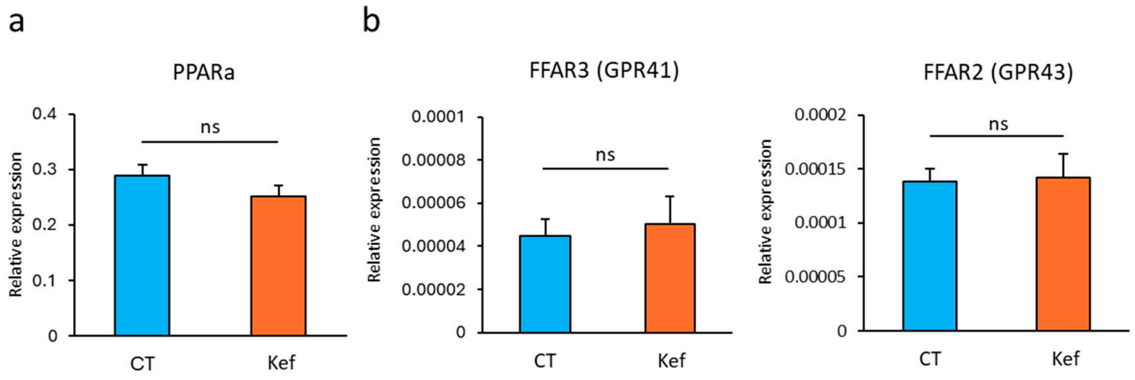

Figure S2. Effect of Kef on expression of gene involved in lipid oxidation and SCFA receptors in the liver. **(a)** RT-PCR analysis of *Ppara* encodoing PPARα in liver tissue (n = 8 - 10). **(b)** RT-PCR analysis of genes encoding SCFA receptor in liver tissue (n = 8 - 10). Kef, rice kefiran; SCFA, short-chain fatty acid.
